# Prevalence and factors associated with HIV viral rebound in individuals on ART: A systematic review study

**DOI:** 10.4102/jphia.v16i1.1324

**Published:** 2025-08-15

**Authors:** Lindokuhle Ndlazi, Mathildah M. Mokgatle, Lindiwe P. Cele, Raikane J. Seretlo

**Affiliations:** 1 Department of Public Health, School of Healthcare Sciences, Sefako Makgatho Health Sciences University, Tshwane, South Africa

**Keywords:** factors, prevalence, antiretroviral therapy, human immune deficiency virus, viral rebound

## Abstract

**Background:**

Viral rebound (VR), the resurgence of detectable human immunodeficiency virus (HIV) viral loads (> 50 copies/mL) after suppression, remains a challenge for individuals on antiretroviral therapy (ART) in South Africa, despite free access to treatment.

**Aim:**

This systematic review aimed to determine the prevalence of HIV VR and its contributing factors among individuals on ART.

**Setting:**

This is a systematic review study, it relies primarily on secondary data, and it does not have a physical setting.

**Method:**

This study is conducted in line with Preferred Reporting Items for Systematic Reviews and Meta-Analyses (PRISMA) guidelines and the Cochrane methodology, and the study was registered with PROSPERO (ID: CRD42024524121). Data were sourced from PubMed, EBSCOhost and Scopus, yielding 89 684 articles. After screening in Rayyan, 23 articles met the inclusion criteria. The risk of bias was assessed using the Joanna Briggs Institute’s (JBI’s) appraisal tool.

**Results:**

Viral rebound varies across different populations. Contributing factors included biological, genetic, demographic, socio-economic and structural elements, as well as incarceration, missed appointments, lifestyle behaviours, travel, multiple sexual partners, ART regimen, age and clinical management. Poor ART adherence emerged as a key driver.

**Conclusion:**

Human immunodeficiency virus viral rebound results from a combination of biological, social and treatment-related factors, with non-adherence to ART being a major contributor. The study highlights the need for improved adherence strategies to reduce VR.

**Contribution:**

This review enhances the understanding of HIV VR prevalence and its contributing factors, while also providing recommendations to mitigate these factors.

## Introduction

South Africa (SA) stands as the nation with the highest number of people living with human immune deficiency virus (HIV) globally.^1^ About 8 million people in the country are HIV-positive, and of all HIV-positive individuals, 62.3% are on antiretroviral therapy (ART).^2^ These numbers of people living with HIV made the country respond by implementing the most extensive ART campaign known as universal test and treat (UTT), which stipulates that regardless of cluster of differentiation 4 (CD4) cell count, all known HIV-positive patients begin first-line ART.^3^

The primary goals of ART are to reduce opportunistic infections and other HIV-associated diseases by maintaining viral suppression.^4^ Moreover, it reduces the chance of treatment resistance, improves the quality of life and is also used as a prophylactic precaution to reduce new infections.^5^ Antiretroviral therapy also reduces the mortality and morbidity caused by acquired immunodeficiency syndrome (AIDS) and HIV and improves people’s quality of life.^5^ Antiretroviral therapy helps to achieve and maintain viral suppression.^5^

Nonetheless, some individuals may experience severe viral rebound (VR) after achieving viral suppression.^4^ Even though ART services are rendered free of charge by the Department of Health (DoH) in SA to the population^1,2^ and despite the implementation of UTT, several factors continue to contribute to the ongoing global issue of HIV VR.^1^ Viral rebound is when two successive viral load measurements within a year show HIV-1 ribonucleic acid (RNA) of 50 copies/mL or > 50 copies/mL, respectively.^6^ There are several factors linked to VR, such as poor adherence,^7^ lower education level,^8^ lower health literacy^9^ and poorer quality of health care, including mistrust of medical providers.^10^ Other factors include substance use and HIV-related stigma,^11^ tuberculosis (TB)^12^ and the type of ART regimen.^13^ Kahabuka et al.^14^ found that participants with advanced HIV illness and shorter clinic visit intervals were more likely to experience VR within the first 2 years of ART.

Several studies have been conducted on HIV VR, and it is evident that VR is still prevalent. Liu et al.^15^ found that out of 3500 patients in the United States (US), 90% of patients had viral suppression, while 10% of patients had VR. Moreover, in Northern Tanzania, Kahabuka et al.^14^ revealed that among the recruited 416 people living with HIV on ART, 40% experienced VR. Maina et al.^7^ conducted a study that showed a high HIV VR rate in Kenya, with Meru having the highest rate (82%), followed by Malindi (81%) and Nakuru (12%). People living with HIV who achieve viral suppression are unable to sustain an undetectable viral load and experience VR, increasing the risk of HIV transmission.^16^ Liu et al.^15^ found that of patients in the US who had 2 years of sustained viral suppression, 10% suffered VR, and 4% experienced prolonged VR. The prevalence of HIV VR occurs in roughly 7.5% of people living with HIV who were able to achieve viral suppression.^16^

Several interventions ensure that people achieve and maintain viral suppression, such as a strategy to achieve sustained post-treatment control that involves eradicating the HIV reservoir or reducing its size so that effective viral reactivation from latency happens infrequently.^17^ Moreover, activating the latent virus and causing the immune system to eliminate infected cells on its own or through spontaneous means is one method of lowering the likelihood of HIV reactivation.^18^

The strategies used in SA to prevent HIV VR switching to second-line ART are defined as moving from non-nucleoside reverse transcriptase inhibitor (NNRTI)-containing ART to protease inhibitor (PI)-containing ART after viremia and intensifying health education,^4^ continuous monitoring of the viral load in people living with HIV is critical for treatment success and preventing transmission^5,19,20,21^ and minimising barriers to access ART.^22^

Even though many studies have been conducted on HIV VR and suppression, little focus has been put on systematic reviews examining the prevalence of VR and the contributing factors among individuals on ART. Hence, this study sought to conduct a systematic review to determine the prevalence of VR and the contributing factors among individuals on ART. Furthermore, the study aimed to identify and address gaps in the field and provide policymakers and stakeholders with new guidelines to support the maintenance of viral suppression once achieved and assist in the implementation of educational programmes that focus and raise awareness on preventing the occurrence of HIV VR among people living with HIV and their caregivers.

## Methods

This review was conducted in accordance with the Preferred Reporting Items for Systematic Reviews and Meta-Analyses (PRISMA) guidelines^23^ and the Cochrane Handbook for Systematic Reviews.^24^ The study was then registered with the International Prospective Register of Systematic Reviews (PROSPERO) on 07 May 2024, under the registration ID CRD42024524121.

### Eligibility criteria

#### Inclusion criteria

- This study included all peer-reviewed published articles that discuss HIV VR globally.
- All English-written articles were included.
- This review included articles published from 2012 onwards that addressed the HIV VR phenomenon.

#### Exclusion criteria

- All non-peer-reviewed, published and non-scholar articles were excluded from the study.
- All articles written in any other language except English were excluded.
- The articles not related to the phenomenon of VR were excluded and articles published in 2011 or earlier were excluded.

### Participants

The researcher used only English-written, peer-reviewed and globally published articles on HIV VR.

### Information sources

The data were sourced from PubMed, EBSCOhost and Scopus. The data search started in March 2024 and ended in May 2024, and to ensure recent data were sourced, the review included studies published from 2012 to 2024.

### Search strategy

**FIGURE 1:**
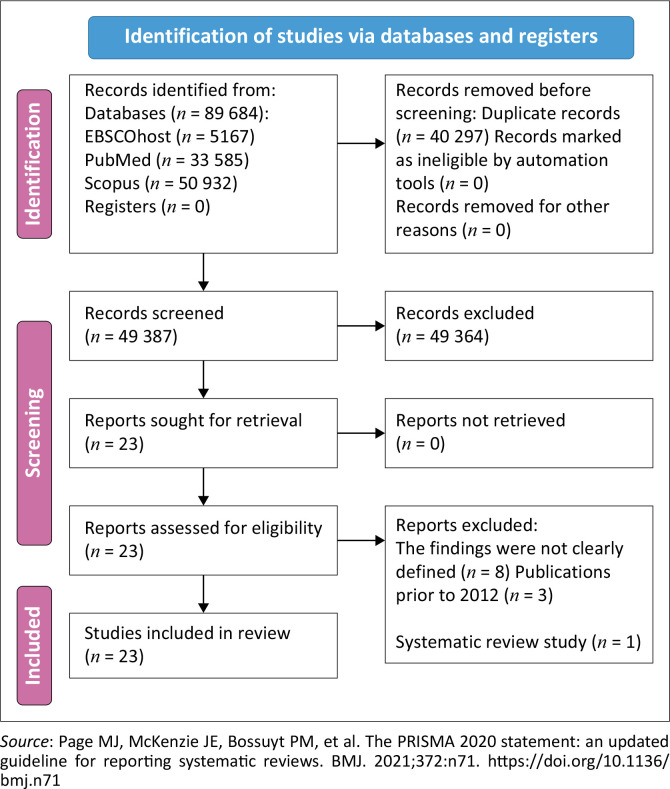
Preferred Reporting Items for Systematic Reviews and Meta-Analyses (PRISMA) diagram illustrating the screening process.

This study mainly focused on secondary data collection, which was retrieved from PubMed, EBSCOhost and Scopus. The data search started in March 2024 and ended in May 2024; the researcher initially collected data on a total of 89 684 articles, of which 33 585 were from PubMed, 5167 were from EBSCOhost and 50 932 were from Scopus (see Figure 1).^25^

The data were searched using the keywords ‘factors’; ‘prevalence’; ‘antiretroviral therapy; antiretroviral’; ‘human immune deficiency virus; ‘highly active antiretroviral therapy’; and ‘viral rebound’. Only English-written, peer-reviewed, globally, sub-Saharan African (SSA) and South African published articles were used; only studies from the year 2012 and above were included; articles were scanned and skimmed; all searched articles were saved in a folder; and any duplicated information was deleted. The main researcher used Rayyan software to include and exclude studies.

### Data collection, selection and extraction process

A total of 89 684 articles were imported into Rayyan for screening, sourced from PubMed (33 585 articles), EBSCOhost (5167 articles) and Scopus (50 932 articles), and the Rayyan software identified 60 201 duplicates, of which 40 297 were deleted and 60 201 were resolved, leading to the exclusion of 47 655 articles. Additionally, 1509 articles remained undecided, while 223 articles were included in the first screening cycle based on their topic relevance. In the second round of exclusions, the main researcher applied the study’s predefined inclusion and exclusion criteria, mainly focusing on the publication year; as a result, all studies published prior to 2012 were excluded from the pool of 1509 undecided articles, reducing the number by 586 and leaving 923 articles for further consideration. Among the 223 articles initially included based on topic relevance, 42 studies were subsequently excluded, so 181 articles remained for further evaluation.

The third round of exclusions focused on assessing the relevance of the articles to the study, which led to the exclusion of 855 articles from the 923 undecided studies, and as a result, 68 articles were retained for inclusion, bringing the total number of included articles to 249 when combined with the 181 articles included in the previous round. In the fourth round, the remaining 249 articles were further analysed, and during this process, 214 articles were excluded, narrowing the selection to 35 articles for eligibility reassessment; after reassessing eligibility, 12 additional articles were excluded because of irrelevance, leaving a final selection of 23 eligible articles (see Figure 1). The selected studies were assessed for eligibility and reviewed with the three supervisors and a conclusion was drawn.

### Quality assessment

All included articles were assessed using Joanna Briggs Institute’s (JBI’s) quality appraisal framework^7^ (see Table 1). All articles included in this systematic review clearly outlined their aims and objectives, methodology, and research design.

**TABLE 1:** Summary table of selected studies.

Author	Title	Objectives	Setting	Design	Findings	JBI-quality appraisal criteria
Aamer et al.^26^	Cells producing residual viremia during antiretroviral treatment appear to contribute to rebound viremia following interruption of treatment	To hypothesise that a reservoir of HIV-infected cells actively produces and releases virions during ART that are potentially infectious and that following ART interruption, these virions can complete full cycles of replication and contribute to rebound viremia.	Seattle, Washington, United States	Longitudinal cohort study	Residual viremia (RV) sequences were identical to pre-ART plasma viruses (5%), infectious viruses induced in quantitative viral outgrowth assays (QVOA) (4%) and rebound viruses (5%) (total *n* = 21/154 [14%] across the three participants). RV sequences identical to ART-interruption ‘rebound’ sequences were detected 0.1–7.3 years prior to ART-interruption. The persisting HIV reservoir produces RV and infectious variants that contribute to rebound viremia upon ART interruption. While our detection of RV that shared identical sequences with replicating viruses detected in QVOA and rebound viruses was not common, these observations support the hypothesis that the infectious reservoir that contributes to rebound viremia also produces RV. The contribution of RV variants to rebound viremia was not associated with whether the clone was detected frequently or rarely among RV sequences. This suggests that multiple or disparate factors likely contribute to RV production and viral rebound or that our sampling was too sparse to detect weak associations.	(Y) Review question clearly and explicitly stated? (Y) Inclusion criteria appropriate for addressing the review question? (Y) Search strategy well-defined and appropriate? (Y) Sources and resources used to search for studies sufficient and relevant? (Y) Criteria for appraising studies clearly defined and appropriate? (Y) Critical appraisal process conducted by at least two independent reviewers? (Y) Measures in place to minimise errors during data extraction? (Y) Any methods used to combine studies appropriate and robust? (Y) Any potential for publication bias assessed and discussed? (Y) Recommendations for policy and/or practice well-supported by the data reported? (Y) Are the directives for future research appropriately detailed and relevant?
Armenia et al.^27^	Very high pre-therapy viral load is a predictor of virological rebound in HIV-1-infected patients starting a modern first-line regimen	To clarify whether high levels of pre-cART viremia are associated with virological rebound after virological suppression.	Rome, Milan, London, Turin, University Hospital, Modena, IRCCS, University Hospital Polyclinic, San Gallicano Dermatological Institute IRCCS, Policlinic Hospital, San Gerardo ‘ Hospital, Monza, Spedali Civili General Hospital, Italy	Retrospective cohort study	Among 5766 patients included, 59.2%, 31.4%, 5.2%, and 4.2% had pre-cART viraemia ≤ 100 000, 100 001–500 000, 500 001–1 000 000, and > 1 000 000 copies/mL, respectively. Patients with pre-cART viraemia levels > 1000 000 copies/mL had the highest probability of VR (> 1000 000; 500 000–1 000 000; 100 000–500 000; < 100 000 copies/mL; VR 50: 28.4%; 24.3%; 17.6%; 13.8%, *p* < 0.0001; VR 200: 14.4%; 11.1%; 7.2%; 7.6%; *p* = 0.009). By Cox multivariable analyses, patients with pre-cART viraemia > 500 000 copies/mL and > 1 000 000 copies/mL showed a significantly higher risk of VR regardless of the VR end point used. No difference in the risk of VR was found between patients with pre-cART viraemia ranging 500 000–1 000 000 copies/mL and those with pre-cART viraemia > 1 000 000 copies/mL, regardless of the VR end point used.	(Y) Review question clearly and explicitly stated? (Y) Inclusion criteria appropriate for addressing the review question? (Y) Search strategy well-defined and appropriate? (Y) Sources and resources used to search for studies sufficient and relevant? (Y) Criteria for appraising studies clearly defined and appropriate? (Y) Critical appraisal process conducted by at least two independent reviewers? (Y) Measures in place to minimise errors during data extraction? (Y) Any methods used to combine studies appropriate and robust? (Y) Any potential for publication bias assessed and discussed? (Y) Recommendations for policy and/or practice well-supported by the data reported? (Y) Are the directives for future research appropriately detailed and relevant?
Burch et al.^28^	Socio-economic status and treatment outcomes for individuals with HIV on antiretroviral treatment in the UK: cross-sectional and longitudinal analyses	To investigate the association of socio-economic factors with antiretroviral therapy (ART) non-adherence, virological non-suppression and virological rebound in HIV-positive people on ART in the UK.	London, Royal Sussex County Hospital, Brighton, UK	Cross-sectional and longitudinal observational study	Low socio-economic status was predictive of longitudinal rebound risk (adjusted hazard ratio [HR] for greatest financial hardship vs. none 2·3, 95% CI: 1·4–3·9; non-employment 3·0, 2·1–4·2; unstable housing vs. homeowner 3·3, 1·8–6·1; non-university education 1·6, 1·1–2·3). Socio-economic disadvantage was strongly associated with poorer HIV treatment outcomes in this setting with universal health care.	(Y) Review question clearly and explicitly stated? (Y) Inclusion criteria appropriate for addressing the review question? (Y) Search strategy well-defined and appropriate? (Y) Sources and resources used to search for studies sufficient and relevant? (Y) Criteria for appraising studies clearly defined and appropriate? (Y) Critical appraisal process conducted by at least two independent reviewers? (Y) Measures in place to minimise errors during data extraction? (Y) Any methods used to combine studies appropriate and robust? (Y) Any potential for publication bias assessed and discussed? (Y) Recommendations for policy and/or practice well-supported by the data reported? (Y) Are the directives for future research appropriately detailed and relevant?
Cole et al.^29^	Extensive characterisation of HIV-1 reservoirs reveals links to plasma viremia before and during analytical treatment interruption	To conduct an extensive analysis on four ART-treated individuals who underwent an analytical treatment interruption (ATI), characterising the proviral genomes and associated integration sites of large, infected clones and phylogenetically linking these to plasma viremia. To investigate the genetic composition and chromosomal location of proviruses within clonally expanded cells and their relationship to rebound viremia.	Belgium, Sydney, Leuven, Seattle, Washington, US	Laboratory-based research study	Our findings confirm that expanded HIV-infected cell clones present in the peripheral blood can contribute to both residual and rebound plasma viremia; the origins of a large fraction of rebounding viruses remain unknown.	(Y) Review question clearly and explicitly stated? (Y) Inclusion criteria appropriate for addressing the review question? (Y) Search strategy well-defined and appropriate? (Y) Sources and resources used to search for studies sufficient and relevant? (Y) Criteria for appraising studies clearly defined and appropriate? (Y) Critical appraisal process conducted by at least two independent reviewers? (Y) Measures in place to minimise errors during data extraction? (Y) Any methods used to combine studies appropriate and robust? (Y) Any potential for publication bias assessed and discussed? (Y) Recommendations for policy and/or practice well-supported by the data reported? (Y) Are the directives for future research appropriately detailed and relevant?
Craw et al.^16^	Viral rebound among persons with diagnosed HIV who achieved viral suppression, United States	To estimate the prevalence of and describe factors associated with viral rebound among adults with diagnosed HIV in the United States who had ≥ 2 viral load tests in a 12-month period.	Atlanta, United States	Cross-sectional study	Among those with ≥ 2 viral load tests who achieved viral suppression, 7.5% demonstrated viral rebound. In multivariable analyses, viral rebound was higher among non-Hispanic black people, persons ages 18–39 years, persons with public insurance, persons recently experiencing homelessness, persons with higher numbers of viral load tests, persons who missed HIV care appointments, and persons with suboptimal adherence to antiretroviral therapy.	(Y) Review question clearly and explicitly stated? (Y) Inclusion criteria appropriate for addressing the review question? (Y) Search strategy well-defined and appropriate? (Y) Sources and resources used to search for studies sufficient and relevant? (Y) Criteria for appraising studies clearly defined and appropriate? (Y) Critical appraisal process conducted by at least two independent reviewers? (Y) Measures in place to minimise errors during data extraction? (Y) Any methods used to combine studies appropriate and robust? (Y) Any potential for publication bias assessed and discussed? (Y) Recommendations for policy and/or practice well-supported by the data reported? (Y) Are the directives for future research appropriately detailed and relevant?
De Scheerder et al.^30^	HIV rebound is predominantly fuelled by genetically identical viral expansions from diverse reservoirs	Conduct an in-depth investigation into the origins of HIV rebound. To determine the source of viral rebound. To further unravel, to what extent specific compartments can act as the source of rebound viruses. To investigate the importance of cellular proliferation in the rebound dynamics. To further characterise the sources of viral rebound during ATI (analytical treatment interruption).	Belgium, Leuven, FD Roosevelt, Sydney	Clinical trail	Shows that viral rebound originates from multiple compartments. We observed a clear link between the rebound viruses and their source in some participants, whereas in others, there were multiple potential contributors or the source was unclear. In participants with groups of identical proviral *env* V1-V3 sequences, our data indicate that these expansions play an important role in viral rebound, confirming that cellular proliferation is a crucial driver of viral persistence, irrespective of the compartment or cell subset. Unique anatomical sources such as gut-associated lymphoid tissue (GALT) can contribute to viral rebound. We did not find clear evidence that the presence of viral production during cART can predict the dynamics of viral rebound; however, it can reveal the presence of dominant clones responsible for viral rebound. Cell proliferation is a driver of viral persistence. Although the origin of the rebound virus is very heterogeneous and diverse, only a small subset of this total pool of infected cells contributes to viremia. Here, we observe the blood, gut, and then LN to contribute to rebound, respectively, from low to high, but individual comparisons between these were not significant (*p* > 0.05).	(Y) Review question clearly and explicitly stated? (Y) Inclusion criteria appropriate for addressing the review question? (Y) Search strategy well-defined and appropriate? (Y) Sources and resources used to search for studies sufficient and relevant? (Y) Criteria for appraising studies clearly defined and appropriate? (Y) Critical appraisal process conducted by at least two independent reviewers? (Y) Measures in place to minimise errors during data extraction? (Y) Any methods used to combine studies appropriate and robust? (Y) Any potential for publication bias assessed and discussed? (Y) Recommendations for policy and/or practice well-supported by the data reported? (Y) Are the directives for future research appropriately detailed and relevant?
Dessie et al.^31^	Modelling viral suppression, viral rebound and state-specific duration of HIV patients with cluster of differentiation 4 (CD4) count adjustment: Parametric multistate frailty model approach	To assess the effects of covariates namely tuberculosis (TB) co-infection, educational status, marital status, age, quality of life (QoL) scores, white and red blood cell parameters, and liver enzyme abnormality on long-term viral suppression, viral rebound, and state-specific duration for HIV-infected individuals before and after treatment.	Durban, South Africa, Ethiopia	Longitudinal observational study	Viral rebound was found to be significantly associated with many sex partners, higher eosinophil count, younger age, lower educational level, higher monocyte counts, having an abnormal neutrophil count, and higher liver enzyme abnormality. Viral suppression was also found to be significantly associated with higher QoL scores and having a stable sex partner.	(Y) Review question clearly and explicitly stated? (Y) Inclusion criteria appropriate for addressing the review question? (Y) Search strategy well-defined and appropriate? (Y) Sources and resources used to search for studies sufficient and relevant? (Y) Criteria for appraising studies clearly defined and appropriate? (Y) Critical appraisal process conducted by at least two independent reviewers? (Y) Measures in place to minimise errors during data extraction? (Y) Any methods used to combine studies appropriate and robust? (Y) Any potential for publication bias assessed and discussed? (Y) Recommendations for policy and/or practice well-supported by the data reported? (Y) Are the directives for future research appropriately detailed and relevant?
Gebreselassie et al.^32^	Ethnicity predicts viral rebound after travel to the tropics in HIV-infected travellers to the tropics in the Swiss HIV cohort study	To describe the traveller population in the Swiss HIV cohort study (SHCS). To determine the frequency of viral rebound in virologically suppressed individuals after a travel episode to the tropics compared to non-travellers.	St. Gallen, Switzerland	Prospective cohort study	Viral rebound was seen in 477 (3.9%) of 12 265 travel episodes and in 5121 (4.5%) of 114 884 non-travel episodes. Compared with European and North American patients, the odds for viral rebound were significantly lower in Southeast Asian (OR: 0.67; 95% CI: 0.51–0.88) and higher in sub-Saharan African (SSA) patients (OR: 1.41; 95% CI: 1.22–1.62). Travel further increased the odds of viral rebound in SSA patients (OR: 2.00; 95% CI: 1.53–2.61).	(Y) Review question clearly and explicitly stated? (Y) Inclusion criteria appropriate for addressing the review question? (Y) Search strategy well-defined and appropriate? (Y) Sources and resources used to search for studies sufficient and relevant? (Y) Criteria for appraising studies clearly defined and appropriate? (Y) Critical appraisal process conducted by at least two independent reviewers? (Y) Measures in place to minimise errors during data extraction? (Y) Any methods used to combine studies appropriate and robust? (Y) Any potential for publication bias assessed and discussed? (Y) Recommendations for policy and/or practice well-supported by the data reported? (Y) Are the directives for future research appropriately detailed and relevant?
Giacomelli et al.^33^	Multidrug-resistant HIV viral rebound during early syphilis: A case report	To highlight the risk of a multidrug-resistant HIV viral rebound during the course of early syphilis even if antiretroviral drug concentrations are within the therapeutic range. Syphilis is an STD that has been associated with an increase in HIV RNA.	Milan, Italy	Case report	Our case suggests that early syphilis can cause an albeit time-limited rebound of HIV viremia in patients with self-reported good adherence to ART and anti-retroviral trough concentrations within the therapeutic range.	(Y) Review question clearly and explicitly stated? (Y) Inclusion criteria appropriate for addressing the review question? (Y) Search strategy well-defined and appropriate? (Y) Sources and resources used to search for studies sufficient and relevant? (Y) Criteria for appraising studies clearly defined and appropriate? (Y) Critical appraisal process conducted by at least two independent reviewers? (Y) Measures in place to minimise errors during data extraction? (Y) Any methods used to combine studies appropriate and robust? (Y) Any potential for publication bias assessed and discussed? (Y) Recommendations for policy and/or practice well-supported by the data reported? (Y) Are the directives for future research appropriately detailed and relevant?
Hayashi et al.^34^	Viral rebound occurrence immediately after drug discontinuation involving neither drug resistance nor latent reservoir	To investigate the viral rebound because of other causes To explore the potential for viral rebound shortly after discontinuing antiviral drug administration. To investigate whether the viral growth rate becomes positive again immediately after drug discontinuation.	Nishi-ku, Japan	Theoretical modelling study	The results suggested that viral rebound is more likely to occur when the processes directly activating the immune responses, that is, the number of effector cytotoxic T lymphocytes (CTLs), are strong compared to those that activate indirectly through immune memory formation. Drugs that effectively suppress the viral growth rate are more likely to lead to rebound. The likelihood of rebound in immunocompromised patients should depend on which processes are impaired in those patients; for example, if the direct activation of CTLs is slower in those patients than in normal hosts, parameter a should be smaller, leading to a lower likelihood of viral rebound. In contrast, if the processes related to memory T-cells are impaired, q and u are smaller, increasing the likelihood of rebound compared to normal hosts. Drug administration may not only reduce the viral growth rate (from *r* to *r* = *r* / [1 + d]). but also impact the rate of memory cell formation (from u to u / (1 + d); this could weaken memory formation more significantly than in cases without side effects, increasing the likelihood of rebounds. Mathematical and numerical analyses revealed that rebound after discontinuation was more likely to occur when the drug effectively reduced viral proliferation, drug discontinuation was delayed, and the processes activating immune responses directly were stronger than those occurring indirectly through immune memory formation.	(Y) Review question clearly and explicitly stated? (Y) Inclusion criteria appropriate for addressing the review question? (Y) Search strategy well-defined and appropriate? (Y) Sources and resources used to search for studies sufficient and relevant? (Y) Criteria for appraising studies clearly defined and appropriate? (Y) Critical appraisal process conducted by at least two independent reviewers? (Y) Measures in place to minimise errors during data extraction? (Y) Any methods used to combine studies appropriate and robust? (Y) Any potential for publication bias assessed and discussed? (Y) Recommendations for policy and/or practice well-supported by the data reported? (Y) Are the directives for future research appropriately detailed and relevant?
Jones et al.^35^	Alcohol, smoking, recreational drug use and association with virological outcomes among people living with HIV: Cross-sectional and longitudinal analyses	To report associations of these factors with antiretroviral therapy (ART) non-adherence, viral non-suppression, and subsequent viral rebound in people living with HIV.	Royal Free Hospital Foundation Trust, London, Brighton, UK	Cross-sectional and longitudinal study	Among 3258 people living with HIV, 2248 (69.0%) were men who have sex with men, 373 (11.4%) were heterosexual men, and 637 (19.6%) were women. A CAGE (C - cut down, A – annoyance, G – guilty, E - eye-opener) score ≥ 2 was found in 568 (17.6%) participants, 325 (10.1%) drank > 20 units/week, 1011 (31.5%) currently smoked, 1242 (38.1%) used recreational drugs and 74 (2.3%) reported injection drug use. In each case, prevalence was much more common among men than among women During follow-up of a subset of 592 people virally suppressed at recruitment, a CAGE score ≥ 2 (adjusted hazard ratio [aHR] = 1.66, 95% CI: 1.03–2.74), use of 3 or more non-injection drugs (aHR = 1.82, 95% CI: 1.12–3.57) and injection drug use (aHR = 2.73, 95% CI: 1.08–6.89) were associated with viral rebound.	(Y) Review question clearly and explicitly stated? (Y) Inclusion criteria appropriate for addressing the review question? (Y) Search strategy well-defined and appropriate? (Y) Sources and resources used to search for studies sufficient and relevant? (Y) Criteria for appraising studies clearly defined and appropriate? (Y) Critical appraisal process conducted by at least two independent reviewers? (Y) Measures in place to minimise errors during data extraction? (Y) Any methods used to combine studies appropriate and robust? (Y) Any potential for publication bias assessed and discussed? (Y) Recommendations for policy and/or practice well-supported by the data reported? (Y) Are the directives for future research appropriately detailed and relevant?
Kang-Birken et al.^36^	HIV viral rebound due to a possible drug-drug interaction between elvitegravir/cobicistat/emtricitabine/tenofovir alafenamide and calcium-containing products: Report of two cases	To report a potential drug-drug interaction in two female patients both receiving treatment for HIV and cerebral toxoplasmosis: one case between E/C/F/TAF with calcium carbonate and a second case involving leucovorin as calcium salt.	Ventura County Medical Centre, Ventura, California, US	Case report	We report a potential drug-drug interaction in two female patients both receiving treatment for HIV and cerebral toxoplasmosis: one case between E/C/F/TAF with calcium carbonate and a second case involving leucovorin as calcium salt. Both cases resulted in a rise in HIV RNA levels and the emergence of the M184 V mutation and resistance to elvitegravir and raltegravir.	(Y) Review question clearly and explicitly stated? (Y) Inclusion criteria appropriate for addressing the review question? (Y) Search strategy well-defined and appropriate? (Y) Sources and resources used to search for studies sufficient and relevant? (Y) Criteria for appraising studies clearly defined and appropriate? (Y) Critical appraisal process conducted by at least two independent reviewers? (Y) Measures in place to minimise errors during data extraction? (Y) Any methods used to combine studies appropriate and robust? (Y) Any potential for publication bias assessed and discussed? (Y) Recommendations for policy and/or practice well-supported by the data reported? (Y) Are the directives for future research appropriately detailed and relevant?
Kankou et al.^37^	Factors associated with virological rebound in HIV-positive sub-Saharan migrants living in France after travelling back to their native country: Agence Nationale de Recherche sur le SIDA-VIH Voyages (ANRS-VIHVO) 2006–2009 study	To determine factors associated with virological rebound on the occasion of a transient stay in the country of origin.	APHP, Bobigny, Lyon, Paris, ORS PACA, Clamart, France, Univ., Paris-Sud, UVSQ	Retrospective cohort study	Among 237 persons (61.6% female), median age 41 years (IQR: 35–47), median time on ART 4.2 years (IQR: 2.2–7.1), 27 (11.4%) experienced VR. Risk factors for virological rebound were male sex, no employment in France, a PI-containing regimen, and self-reported non-adherence during the stay abroad. In models not including self-reported adherence, unexpected extensions of the stay and travelling during Ramadan while respecting the fast were associated with virological rebound.	(Y) Review question clearly and explicitly stated? (Y) Inclusion criteria appropriate for addressing the review question? (Y) Search strategy well-defined and appropriate? (Y) Sources and resources used to search for studies sufficient and relevant? (Y) Criteria for appraising studies clearly defined and appropriate? (Y) Critical appraisal process conducted by at least two independent reviewers? (Y) Measures in place to minimise errors during data extraction? (Y) Any methods used to combine studies appropriate and robust? (Y) Any potential for publication bias assessed and discussed? (Y) Recommendations for policy and/or practice well-supported by the data reported? (Y) Are the directives for future research appropriately detailed and relevant?
Ladak et al.^38^	Substance use patterns and HIV-1 RNA viral load rebound among HIV-positive illicit drug users in a Canadian setting	To investigate the contribution of substance, use patterns on rates of VL rebound.	Alberta, Edmonton, AB, British Columbia Centre, St. Paul’s Hospital, Vancouver, BC, Population, Canada	Prospective cohort study	Over follow-up, 198 (35.1%) participants experienced ≥ one instance of VL rebound. In adjusted analyses, VL rebound was associated with younger age (adjusted hazard ratio [AHR] = 0.97, 95% CI: 0.95, 0.98), heroin injection (≥ daily vs. < daily, AHR = 1.52, 95% CI: 1.01, 2.30), crack use (≥ daily vs. < daily, AHR = 1.73, 95% CI: 1.08, 1.92), and heavy alcohol use (≥ four vs. < four drinks/day, AHR = 1.97, 95% CI: 1.17, 3.31).	(Y) Review question clearly and explicitly stated? (Y) Inclusion criteria appropriate for addressing the review question? (Y) Search strategy well-defined and appropriate? (Y) Sources and resources used to search for studies sufficient and relevant? (Y) Criteria for appraising studies clearly defined and appropriate? (Y) Critical appraisal process conducted by at least two independent reviewers? (Y) Measures in place to minimise errors during data extraction? (Y) Any methods used to combine studies appropriate and robust? (Y) Any potential for publication bias assessed and discussed? (Y) Recommendations for policy and/or practice well-supported by the data reported? (Y) Are the directives for future research appropriately detailed and relevant?
Maina et al.^7^	Incidences and factors associated with viral suppression or rebound among HIV patients on combination antiretroviral therapy from three counties in Kenya	To investigate the incidence rates of viral rebound following viral suppression, factors associated with viral rebound, and the durability of viral suppression among HIV-infected individuals on ART from Kilifi, Meru, and Nakuru counties in Kenya.	Nairobi, Kenya	Retrospective cohort study	The overall viral rebound rate was 41%, with site-specific viral rebound of 88.2%, 18.6%, and 18.0% in Nakuru, Malindi, and Meru, respectively. There was an overall rate of first viral rebound of 3.9 (95% CI: 6.9–14.4), 0.7 (95% CI: 0.5–1.0), and 0.89 (95% CI: 0.64–1.24) per 100 person-months in Nakuru, Malindi, and Meru, respectively. Good ART adherence (*p* = 0.0002), widow status (*p* = 0.0062), and World Health Organization (WHO) stage I (*p* = 0.0002) were associated with viral suppression, while poor ART adherence (*p* < 0.0001), WHO stage II (*p* = 0.0024), and duration on ART of 36 months (*p* = 0.0350) were associated with viral rebound.	(Y) Review question clearly and explicitly stated? (Y) Inclusion criteria appropriate for addressing the review question? (Y) Search strategy well-defined and appropriate? (Y) Sources and resources used to search for studies sufficient and relevant? (Y) Criteria for appraising studies clearly defined and appropriate? (Y) Critical appraisal process conducted by at least two independent reviewers? (Y) Measures in place to minimise errors during data extraction? (Y) Any methods used to combine studies appropriate and robust? (Y) Any potential for publication bias assessed and discussed? (Y) Recommendations for policy and/or practice well-supported by the data reported? (Y) Are the directives for future research appropriately detailed and relevant?
Megasari and Wijaksa,^39^	Factors affecting HIV viral load of antiretroviral therapy-experienced and naive individuals residing in Bali, Indonesia	To measure HIV VL and identify associated factors, in people living with HIV experienced and naive to ART, residing in Buleleng, Bali, Indonesia.	Bali, Indonesia	Cross-sectional study	The presence of major HIV drug resistance mutations (HIVDRMs) and the number of ARV affected significantly contributed to higher VL among ART-experienced individuals. Tuberculosis (TB) co-infection, body mass index (BMI), and WHO clinical stage were significantly causing higher VL among ART-naive individuals.	(Y) Review question clearly and explicitly stated? (Y) Inclusion criteria appropriate for addressing the review question? (Y) Search strategy well-defined and appropriate? (Y) Sources and resources used to search for studies sufficient and relevant? (Y) Criteria for appraising studies clearly defined and appropriate? (Y) Critical appraisal process conducted by at least two independent reviewers? (Y) Measures in place to minimise errors during data extraction? (Y) Any methods used to combine studies appropriate and robust? (Y) Any potential for publication bias assessed and discussed? (Y) Recommendations for policy and/or practice well-supported by the data reported? (Y) Are the directives for future research appropriately detailed and relevant?
Min et al.^8^	Evaluating HIV viral rebound among persons on suppressive antiretroviral treatment in the era of ‘undetectable equals untransmittable (U = U)’	To demonstrate that persons with HIV maintaining viral suppression do not transmit HIV to HIV-negative partners through condomless sex, leading to the ‘Undetectable = Untransmittable (U = U)’.	Rhode Island, US	Retrospective cohort study	A total of 1242 patients with viral suppression were included in the baseline cohort. In each follow-up year, 85% – 90% maintained viral suppression, 2% – 5% experienced viral rebound, and 8% – 10% had a gap in VL monitoring. In the logistic regression models, retention in care was significantly associated with viral suppression, while younger age, black race, high school or equivalent education, non-men who have sex with men, and history of incarceration were significantly associated with viral rebound.	(Y) Review question clearly and explicitly stated? (Y) Inclusion criteria appropriate for addressing the review question? (Y) Search strategy well-defined and appropriate? (Y) Sources and resources used to search for studies sufficient and relevant? (Y) Criteria for appraising studies clearly defined and appropriate? (Y) Critical appraisal process conducted by at least two independent reviewers? (Y) Measures in place to minimise errors during data extraction? (Y) Any methods used to combine studies appropriate and robust? (Y) Any potential for publication bias assessed and discussed? (Y) Recommendations for policy and/or practice well-supported by the data reported? (Y) Are the directives for future research appropriately detailed and relevant?
Mujugira et al.^40^	Younger age predicts failure to achieve viral suppression and virologic rebound among HIV-1-infected persons in serodiscordant partnerships	Previously found that younger age and higher CD4 counts were associated with delayed initiation of ART by HIV-1-infected partners in serodiscordant partnerships. To explore whether those same factors were associated with failure to achieve viral suppression.	Seattle, Atlanta, Georgia, Winnipeg, Canada, Nairobi, Kenya, Washington	Prospective cohort study	Younger age (adjusted hazard ratio [aHR] 1.05 for every 5 years younger; *p*= 0.006), lower pretreatment CD4 count (aHR 1.26; *p* = 0.009 for ≤ 250 compared with > 250 cells/μL), and higher pretreatment HIV-1 RNA quantity (aHR 1.21 per log10; *p* < 0.001) independently predicted failure to achieve viral suppression. Following initial viral suppression, 8.8% (*n* = 76/867) experienced virologic rebound (HIV-1 RNA > 200 copies/mL): 6.3% and 11.5% by 6 and 12 months after initial suppression, respectively. Age was the only factor associated with increased risk of virologic rebound (aHR 1.33 for every 5 years younger; *p* = 0.005).	(Y) Review question clearly and explicitly stated? (Y) Inclusion criteria appropriate for addressing the review question? (Y) Search strategy well-defined and appropriate? (Y) Sources and resources used to search for studies sufficient and relevant? (Y) Criteria for appraising studies clearly defined and appropriate? (Y) Critical appraisal process conducted by at least two independent reviewers? (Y) Measures in place to minimise errors during data extraction? (Y) Any methods used to combine studies appropriate and robust? (Y) Any potential for publication bias assessed and discussed? (Y) Recommendations for policy and/or practice well-supported by the data reported? (Y) Are the directives for future research appropriately detailed and relevant?
Offersen et al.^41^	HIV antibody Fc N-linked glycosylation is associated with viral rebound	To identify features that are tracked most significantly with viral rebound kinetics. To define whether specific glycan profiles could be associated with viral rebound. To identify a minimal set of antibody functions associated with time to rebound. To define whether specific glycan profiles could be associated with viral rebound. To explore whether a unique functional antibody profile may track selectively with differential viral rebound time. To further define whether specific biophysical humoral changes contributed to the observed functional associations, we next sought to characterise the Fc-linked biophysical changes in HIV-specific antibodies across subjects that are tracked with rebound kinetics. To explore the biological basis for the link between the G2 signature and viral rebound.	Cambridge, Denmark, US, Baltimore, Boston, Hospital, Massachusetts General Hospital, Hamburg	Clinical trial, observational study	The Gp120-specific Fc-glycan profile was also associated with time to rebound in 2 additional separate HIV cohorts. Env-specific G2 glycan levels at the time of treatment discontinuation (v12) were significantly associated with increased time to viral rebound. HIV Env-specific sialylated G2 glycans were found to be associated with viral rebound across three different interventional studies, suggesting that these antibody profiles are independent of the latency reversal approach used. Data suggests that changes in HIV Env-specific Antibody Dependent Complement Deposition (ADCD) and Activity-Dependent Neuroprotective Protein (ADNP) during the PNB treatment period were linked with a longer time to viral rebound, unrelated to CD4 counts, CD4 nadir, or transient viremia. The glycans are strongly linked with time to viral rebound. Non-bisecting variants of the G2 glycan are all associated with a longer time to rebound. Bisecting, G0 and G1 glycan structures were associated with more rapid rebound.	(Y) Review question clearly and explicitly stated? (Y) Inclusion criteria appropriate for addressing the review question? (Y) Search strategy well-defined and appropriate? (Y) Sources and resources used to search for studies sufficient and relevant? (Y) Criteria for appraising studies clearly defined and appropriate? (Y) Critical appraisal process conducted by at least two independent reviewers? (Y) Measures in place to minimise errors during data extraction? (Y) Any methods used to combine studies appropriate and robust? (Y) Any potential for publication bias assessed and discussed? (Y) Recommendations for policy and/or practice well-supported by the data reported? (Y) Are the directives for future research appropriately detailed and relevant?
Opoku et al.^42^	Factors associated with viral suppression and rebound among adult HIV patients on treatment: A retrospective study in Ghana	To evaluate viral suppression and rebound and their associated factors among adult patients on ART in Kumasi, Ghana.	Kumasi, Ghana	Retrospective cohort study	Proportions of patients with viral suppression and viral rebound were 76.1% and 21.0%, respectively. Being diagnosed at WHO stage I (aOR = 11.40, 95% CI: 3.54–36.74, *p* < 0.0001), having good adherence to ART (aOR = 5.09, 95% CI: 2.67–9.73, *p* < 0.0001), taking a nevirapine-based regimen (aOR = 4.66, 95% CI: 1.20–18.04, *p* = 0.0260), and increasing duration of treatment (*p* < 0.0001) were independently associated with higher odds of viral suppression. However, being diagnosed at WHO stage II (aOR = 7.39, 95% CI: 2.67–20.51; *p* < 0.0001) and stage III (aOR = 8.62, 95% CI: 3.16–23.50; *p* < 0.0001), having poor adherence (aOR = 175.48, 95% CI: 44.30–695.07; *p* < 0.0001), recording baseline suppression value of 20–49 copies/mL (aOR = 6.43, 95% CI: 2.72–15.17; *p* < 0.0001), and being treated with Zidovudine/Lamivudine/Efavirenz (aOR = 6.49, 95% CI: 1.85–22.79; *p* = 0.004) and zidovudine/lamivudine/nevirapine (aOR = 18.68, 95% CI: 1.58–220.90; *p* = 0.02) were independently associated with higher odds of viral rebound.	(Y) Review question clearly and explicitly stated? (Y) Inclusion criteria appropriate for addressing the review question? (Y) Search strategy well-defined and appropriate? (Y) Sources and resources used to search for studies sufficient and relevant? (Y) Criteria for appraising studies clearly defined and appropriate? (Y) Critical appraisal process conducted by at least two independent reviewers? (Y) Measures in place to minimise errors during data extraction? (Y) Any methods used to combine studies appropriate and robust? (Y) Any potential for publication bias assessed and discussed? (Y) Recommendations for policy and/or practice well-supported by the data reported? (Y) Are the directives for future research appropriately detailed and relevant?
Palmer et al.^43^	Viral suppression and viral rebound among young adults living with HIV in Canada	To describe the prevalence and covariates of viral suppression and subsequent rebound among younger (≤ 29 years old) compared with older adults.	Vancouver, Canada	Retrospective cohort study	In a large Canadian HIV treatment cohort, we found that younger adults were less likely to achieve viral suppression compared with older adults. Among younger adults, sex, era of cART initiation, history of IDU, composition of first cART regimen and viral load were independently associated with viral suppression, in addition to the characteristics, being Indigenous and CD4 cell count at cART initiation were associated with viral rebound. The UK Collaborative HIV Cohort Study (UK-CHIC) found that for every 10-year increase in age, the rate of viral rebound decreased by 28%,^14^ compared to a 1% decrease per year in our analysis. A large adolescent and young adult cohort in the United States (REACH) found that only 51% of young people maintained a suppressed viral load for a year;^23^ in contrast our study indicated that only 11% of youth experienced viral rebound at 12 months post suppression. Our results align with previous research indicating that young women are at greater risk of viral rebound compared to young men, which may be partially explained by lower levels of adherence among women. We found that unboosted PIs were associated with a reduced likelihood of achieving viral suppression and increased risk for viral rebound. This may be because of unboosted PI regimens being more complex compared to NNRTI. Our finding that younger adults who identify as Indigenous are more likely than non-Indigenous people to experience viral rebound suggests the importance of retention in care and follow-up while on treatment. The independent associates of viral suppression and rebound for younger adults are similar to those known to affect older adults (e.g. history of IDU), highlighting at-risk populations for future research and intervention.	(Y) Review question clearly and explicitly stated? (Y) Inclusion criteria appropriate for addressing the review question? (Y) Search strategy well-defined and appropriate? (Y) Sources and resources used to search for studies sufficient and relevant? (Y) Criteria for appraising studies clearly defined and appropriate? (Y) Critical appraisal process conducted by at least two independent reviewers? (Y) Measures in place to minimise errors during data extraction? (Y) Any methods used to combine studies appropriate and robust? (Y) Any potential for publication bias assessed and discussed? (Y) Recommendations for policy and/or practice well-supported by the data reported? (Y) Are the directives for future research appropriately detailed and relevant?
Small et al.^44^	Plasma HIV-1 RNA viral load rebound among people who inject drugs receiving antiretroviral therapy (ART) in a Canadian setting: An ethno-epidemiological study	To investigate the circumstances surrounding the emergence of rebound episodes among people who inject drugs in Vancouver, BC, Canada.	St. Paul’s Hospital, Burrard Street, Vancouver, Canada, Burnaby, British Columbia Centre, South Wales, Sydney	Mixed-methods study	Viral rebound episodes were shaped by the interplay between various individual, social, and environmental factors that disrupted routines facilitating adherence. Structural-environmental influences resulting in non-adherence included housing transitions, changes in drug use patterns and intense drug scene involvement, and inadequate care for co-morbid health conditions. Social-environmental influences on ART adherence included poor interactions between care providers and patients producing non-adherence, and understandings of HIV treatment that fostered intentional treatment discontinuation.	(Y) Review question clearly and explicitly stated? (Y) Inclusion criteria appropriate for addressing the review question? (Y) Search strategy well-defined and appropriate? (Y) Sources and resources used to search for studies sufficient and relevant? (Y) Criteria for appraising studies clearly defined and appropriate? (Y) Critical appraisal process conducted by at least two independent reviewers? (Y) Measures in place to minimise errors during data extraction? (Y) Any methods used to combine studies appropriate and robust? (Y) Any potential for publication bias assessed and discussed? (Y) Recommendations for policy and/or practice well-supported by the data reported? (Y) Are the directives for future research appropriately detailed and relevant?
Stöhr et al.^45^	Factors associated with virological rebound in HIV-infected patients receiving protease inhibitor monotherapy	To identify factors associated with the risk of viral load rebound.	Kingsway, United Kingdom	Randomised controlled trial (RCT)	Of 290 participants initiating protease inhibitor monotherapy (80% darunavir, 14% lopinavir, and 6% others), 93 developed viral loads rebound on monotherapy. The risk of viral load rebound peaked at 9 months after starting monotherapy and then declined to approximately 5 per 100 person-years from 18 months onwards. Independent predictors of viral load rebound were duration of viral load suppression before starting monotherapy (hazard ratio 0.81 per additional year < 50 copies/mL; *p* < 0.001), CD4+ cell count (hazard ratio 0.73 per additional 100 cells/mL for CD4+ nadir; *p* = 0.008), ethnicity (hazard ratio 1.87 for non-white versus white, *p* = 0.025), but not the protease inhibitor agent used (*p* = 0.27). Patients whose viral load were analysed with the Roche TaqMan-2 assay had a 1.87-fold risk for viral load rebound compared with the Abbott real-time assay (*p* = 0.012).	(Y) Review question clearly and explicitly stated? (Y) Inclusion criteria appropriate for addressing the review question? (Y) Search strategy well-defined and appropriate? (Y) Sources and resources used to search for studies sufficient and relevant? (Y) Criteria for appraising studies clearly defined and appropriate? (Y) Critical appraisal process conducted by at least two independent reviewers? (Y) Measures in place to minimise errors during data extraction? (Y) Any methods used to combine studies appropriate and robust? (Y) Any potential for publication bias assessed and discussed? (Y) Recommendations for policy and/or practice well-supported by the data reported? (Y) Are the directives for future research appropriately detailed and relevant?

Note: Scale: y = yes, p = poor, ni = not indicated. Please see full reference list of this article, Ndlazi L, Mokgatle MM, Cele LP, Seretlo RJ. Prevalence and factors associated with HIV viral rebound in individuals on ART: A systematic review study. J Public Health Africa. 2025;16(1), a1324. https://doi.org/10.4102/jphia.v16i1.1324, for more information.

HIV, human immunodeficiency virus; ART, antiretroviral therapy; VR, viral rebound; OR, odds ratio; CI, confidence interval; IQR, interquartile range; JBI, Joanna Briggs Institute; cART, combined antiretroviral treatment; LN, lymph node; TB, tuberculosis; CD4, cluster of differentiation 4; STD, Sexually transmitted disease; RNA, ribonucleic acid; UK, United Kingdom; US, United States; APHP, Assistance Publique-Hôpitaux de Paris; ORS PACA, Observatoire Régional de la Santé Provence-Alpes-Côte d’Azur; UVSQ, Université de Versailles Saint-Quentin-en-Yvelines; AB, Alberta; BC, British Columbia; PI, protease inhibitor; VL, viral load; IDU, injection drug use; NNRTI, non-nucleoside reverse transcriptase inhibitor.

### Ethical considerations

This study used secondary data; however, Sefako Makgatho Health Sciences Ethics Committee (SMUREC) (Reference Number: SMUREC/H/464/2023: PG) provided ethical clearance and permission for the project. The systematic review was registered on PROSPERO (International Register of Systematic Reviews; CRD42024524121).

## Results

Among the 23 studies included in this review, 13 studies discuss the prevalence of HIV VR. The findings across these studies indicate considerable variability in VR prevalence, influenced by factors such as population characteristics, geographic region and ART regimens.

From the analysis of the table presented in the studies, it is evident that genetics and biological factors emerge as the most significant drivers of HIV VR. Genetic predispositions, along with factors such as drug–drug interactions (DDIs), co-infections and medication interactions, contribute substantially to the persistence of the virus and its ability to rebound after treatment interruption. These biological factors remain central to understanding the dynamics of HIV VR, as they directly influence the virus’s ability to persist in the body, even under ART.

In addition, socio-economic and structural barriers are identified as the second-largest contributors to HIV VR. Factors such as low socio-economic status, unstable housing, lack of access to health care and incarceration significantly impact an individual’s ability to adhere to treatment regimens and attend necessary follow-up appointments. These structural challenges create an environment that makes effective HIV management more difficult, thereby increasing the risk of VR.

Demographic factors, particularly age, also play a critical role in determining the likelihood of HIV VR. Research consistently shows that younger age is associated with a higher risk of VR, potentially because of lower adherence rates, less consistent engagement in care and possibly immune system dynamics that differ from older populations. This underscores the need for targeted interventions for younger individuals, as they may face unique challenges in managing HIV effectively.

Additionally, behavioural and lifestyle factors are found to contribute to the risk of HIV VR significantly. Increased likelihood of VR has been associated with substance use, including alcohol and drug abuse, as well as high-risk behaviours such as having multiple sexual partners or engaging in travel during ART treatment, have all been linked to. These factors disrupt the consistency of ART adherence and can impair immune system function, further complicating the management of HIV.

Finally, treatment and clinical management factors, particularly poor adherence to ART, are significant drivers of HIV VR. Non-adherence, whether because of forgetfulness, dissatisfaction with treatment regimens, or other personal and logistical barriers, remains among the most substantial contributors to VR. The ability to effectively manage ART, including ensuring proper drug regimen adherence and timely follow-up appointments, is crucial in preventing the occurrence of VR.

Therefore, the drivers of HIV VR are multifaceted, involving a complex interplay of genetic, socio-economic, demographic, behavioural and clinical management factors. Understanding these diverse influences is essential for developing comprehensive strategies aimed at reducing the risk of HIV VR and improving long-term treatment outcomes.

### Characteristics of selected studies

Table 1 provides a summary of selected studies in this review. This review included 23 studies based on predefined selection criteria. The studies were sourced from three academic data sources: 15 from PubMed, 8 from Scopus and 1 from EBSCOhost. Most studies are non-African published, representing 83% of the sample. Thirteen per cent of studies are from SSA and 4% of studies originate from SA. The 4% of studies on HIV VR in SA indicate a notable gap in research on this topic over the past decade.

The studies included in this review were published between 2016 and 2024. The distribution of publications across years is as follows: 30.43% of studies were published in 2020, 21.74% of studies were published in 2019 and 17.39% of studies were published in 2016. Among the remaining publications, 13.04% were published in 2022, with an additional 4.35% published in each of the years 2017, 2018, 2023, and 2024.

The studies in this review employed a range of methodological approaches. The majority were retrospective cohort studies, with six studies using this design. Three prospective cohort studies followed this, and two cross-sectional studies and two case reports. Other study designs included one each of the following: clinical trial, clinical trial and observational study, cross-sectional and longitudinal study, longitudinal study, laboratory-based study, longitudinal cohort study, longitudinal observational study, mixed-methods, randomised controlled trial and theoretical model study.

The PRISMA diagram (Figure 1) illustrates the screening process followed in this study for HIV VR research, thus ensuring transparency.

## Discussion

Research consistently demonstrates that the prevalence of HIV VR remains a significant public health concern, with notable variability in prevalence across different populations. These findings align with those of previous studies, such as the research by Rosen et al.^46^ which reported that only 2.5% of people living with HIV experienced VR. In a study by Bansi et al.^47^ higher rates of VR were observed in individuals who had previously discontinued treatment, with a rate ratio of 1.64 (1.43–1.88), and the total VR rate for every 100 person-years was calculated at 8.07 (7.78–8.36).

Similarly, research by Bridges et al.^48^ found that the cumulative incidence of VR 1 year after achieving viral suppression varied across different ethnic groups, with 13% of Indigenous Caucasian individuals; 7% of African, Caribbean and black (ACB) individuals; and 5% of both other ethnic groups and individuals of unknown ethnicity experiencing rebound. However, a study by Grabowski et al.^49^ showed a significant reduction in VR and chronic viremia with the implementation of UTT strategies. In their research, Grabowski et al.^49^ discovered that the prevalence of VR decreased from 4.4% to 2.7%, while chronic viremia dropped from 20.8% to 13.3%. These studies highlight the ongoing challenge of managing VR and underscore the need for tailored approaches to HIV treatment that account for demographic, treatment and health care-related factors.

Research has revealed that HIV VR is genetically linked, which is consistent with findings from Cole et al.^29^ that show that latently infected cells can become activated and produce the virus once ART is discontinued. Vallejo et al.^50^ demonstrated that the TLR9-1635AA genotype is independently associated with an increased risk of HIV VR following the cessation of ART.

However, not all studies support the view that genetic factors are pivotal in HIV VR. Mpolya^1^ highlighted that patient-specific variables and treatment adherence may significantly influence VR. Similarly, Thorball et al.^51^ found no meaningful correlation between human genetic variation and the size of the HIV reservoir or its rate of decay after suppressive ART, which raises questions about the role of genetics in the dynamics of HIV VR. These mixed findings highlight the complexity of the relationship between host genetics and HIV VR, suggesting that further investigation is needed to understand better the extent to which genetic factors contribute to VR.

The findings demonstrate a strong association between the type of ART regimen and HIV VR. Childs et al.^52^ suggest that the choice of first-line ART influences the outcomes following VR. Furthermore, Liu et al.^15^ found that individuals on integrase strand transfer inhibitors (INSTIs) exhibited a higher likelihood of VR. In contrast, Boucoiran et al.^53^ found no discernible correlation between the type of ART regimen and HIV VR, implying that ART regimen choice may not significantly affect the incidence of VR.

The study finds that HIV VR is associated with DDIs and drug resistance. This finding is consistent with previous research by Rock et al.^54^ Patients taking INSTIs such as bictegravir may experience VR because of DDIs with divalent cations like calcium and zinc. Furthermore, Kumar et al.^55^ stated that DDIs, particularly those affecting the induction or inhibition of the cytochromes P450 (CYP) enzyme, can reduce the bioavailability of ART, thereby increasing viral load and diminishing the efficacy of ART, and this can result in HIV VR in affected individuals.

However, Iniesta-Navalón et al.^56^ reported that DDIs did not significantly impact viral load outcomes in their observed patient population. These findings underscore the complexity of HIV VR, highlighting the interplay between drug resistance, DDIs and immune response in determining viral outcomes. While DDIs and drug resistance may contribute to VR, immune factors also play a pivotal role in viral dynamics.

The study found that co-infections, particularly TB and syphilis, are significant contributors to VR in people living with HIV, which is consistent with findings from another research. For instance, co-infections such as acute malaria and active TB also contribute to VR, with acute malaria associated with a 0.67 log_10_ increase and active TB linked to a 0.40 log_10_ rise in HIV viral load.^57^ Additionally, the presence of multiple viral infections may create complex interactions at the cellular level, which can enhance HIV replication and contribute to VR.^58^ These dynamics highlight the need for effective management of co-infections to prevent VR and slow HIV progression.

The study finds that HIV VR is associated with low socioeconomical status (SES), which is consistent with the findings from previous research. Abgrall et al.^59^ linked a higher risk of VR to low educational attainment, poor financial conditions and non-disclosure of HIV status. Liu et al.^15^ stated that SES, employment and food security are risk factors for VR. However, López et al.^60^ found that SES was insignificant in HIV VR. This divergence highlights the complexity of the relationship between socio-economic factors and HIV VR, suggesting that further research is needed to clarify their precise role and impact.

The study identifies a history of incarceration as a significant factor contributing to VR, a finding that aligns with prior research. Periods of incarceration often disrupt individuals’ ART regimens, leading to breaks in therapy that are correlated with a heightened risk of VR.^61^ Similarly, Westergaard et al.^62^ emphasised that incarceration is linked to poorer treatment outcomes, further highlighting the detrimental effects of incarceration on HIV care and viral suppression. These disruptions underscore the challenges faced by individuals transitioning in and out of incarceration, which can undermine the effectiveness of ART and contribute to VR.

The study finds that VR is strongly associated with patient loss to follow-up, which aligns with several prior studies. Hao^63^ reported higher rates of VR among youth with HIV who missed appointments compared to those who remained engaged in regular care. Additionally, a study by Sethi et al.^64^ showed that the risk of VR, often coupled with clinically significant resistance, was independently associated with missed clinic visits.

The research indicates that alcohol and substance use are significant factors contributing to the development of HIV VR. These findings are consistent with those of Tanner et al.^65^ who found an association between VR and injectable drug use. At the same time, Liu et al.^15^ found that smoking is associated with VR. However, Wynn et al.^66^ observed a negative correlation between alcohol use and adherence to ART, although this association was not statistically significant in terms of viral load suppression.

The study highlights a significant association between HIV VR and travel, aligning with other researchers’ findings. Travel can impede adherence to ART in people living with HIV, leading to ART interruptions and an increased risk of VR.^67^ However, Joos et al.^68^ suggested that travel itself may not be the primary cause of VR, as it is typically because of the reactivation of latent HIV reservoirs rather than sustained low-level replication. This discourse reflects the complexity of the relationship between travel and HIV VR, suggesting that while travel may be a contributing factor, other underlying biological mechanisms must also be considered.

The study finds that having multiple sexual partners and engaging in risky sexual behaviour contribute to HIV VR, which aligns with the findings of other researchers. Risk compensation behaviours where individuals engage in riskier sexual practices because they believe ART will protect them have been reported to increase the risk of sexually transmitted infections (STIs) and VR.^69^

Therefore, while multiple and concurrent sexual partnerships are a key factor in the spread of HIV, more research is needed to fully understand the interplay between sexual behaviour, ART adherence and VR. These findings highlight the multifaceted nature of HIV transmission, emphasising the need for targeted prevention strategies that address both behavioural risks and the challenges of maintaining viral suppression.

The study identifies poor adherence or non-compliance as a key factor contributing to HIV VR, a finding consistent with previous research. Achappa et al.^70^ reported that VR, continued immunosuppression and viral resistance can result from non-adherence to ART. However, Maggiolo et al.^71^ suggested that while mild non-adherence to ART may not necessarily result in virologic failure or VR, it is often associated with low-level residual viremia. These findings underscore the critical role of consistent adherence in preventing virologic failure and ensuring long-term viral suppression.

The study finds that HIV VR is associated with pre-ART conditions and HIV staging, which aligns with findings from previous research. For instance, Castagna et al.^72^ observed a significant correlation between pre-ART factors and the magnitude of VR. According to Robertson et al.^73^ VR is more likely in individuals who had an initial CD4 count ≥ 350 at diagnosis or those diagnosed with AIDS. Similarly, Ceccherini-Silberstein et al.^74^ reported that a higher level of pre-ART HIV deoxyribonucleic acid (DNA) was associated with an increased likelihood of VR.

This study identifies gender as a contributing factor to HIV VR, which aligns with several other research findings. In particular, several studies have suggested that women are less likely than men to experience HIV VR.^75^ However, Adams et al.^76^ found no significant correlation between female sex and the occurrence of HIV VR. Furthermore, men who have sex with men (MSM) born in France did not confer a higher risk of HIV VR compared to heterosexual individuals born in France.^59^ Suggesting that gender alone is not a determining factor in VR risk. Thus, these studies collectively highlight the complexity of gender as a variable in HIV VR, with conflicting results that warrant further investigation.

The study finds that black individuals are disproportionately affected by VR, a finding that aligns with several previous studies. For instance, Dhairyawan et al.^77^ reported that Asian and black populations were more likely to experience VR compared to white individuals, further underscoring racial disparities in HIV outcomes. Similarly, Liu et al.^15^ identified race as a significant factor associated with HIV-related VR. Notably, Liu et al.^15^ also observed that among people living with HIV who had previously achieved sustained viral suppression, race remained correlated with the likelihood of experiencing VR. Collectively, these findings suggest that black individuals may be particularly vulnerable to VR. However, while race appears to be an important factor, it is essential to acknowledge that broader social, behavioural and medical determinants also contribute to these disparities.

Studies have shown that HIV VR is associated with younger age, a finding consistent with^11,13,14,15,16,17^ all of who identified a higher likelihood of HIV VR in younger individuals compared to older populations. The independent correlates of viral suppression and VR for younger adults are similar to those affecting older individuals, suggesting that age alone may not fully account for variations in VR outcomes.

### Strength and limitations

One of the primary strengths of this systematic review was its rigorous methodology, following a structured and transparent process for identifying, selecting and synthesising studies. Using multiple data sources like PubMed, Scopus and EBSCOhost ensured a broad scope and minimised bias. The systematic approach to study selection and data extraction increased the reliability of findings, while including diverse studies provided a general understanding of HIV VR. Narrative synthesis and the PRISMA flow diagram enhanced clarity and assessing study quality and risk of bias ensured conclusions were based on strong evidence, offering insights into HIV VR prevalence and contributing factors.

However, several limitations should be noted. The review was restricted to studies in English, potentially introducing language bias. Some studies lacked detailed reporting on key variables, limiting full validity evaluation. Only Department of Higher Education and Training (DHET) accredited journals were included, excluding grey literature and unpublished studies, and strict inclusion criteria limited studies to those published from 2012 onwards. These factors may have caused publication bias and incomplete findings on HIV VR.

### Recommendations

Health care providers should be trained to recognise and address HIV VR factors, including ART non-adherence, interruptions and suboptimal regimens, using evidence-based information to enhance personalised care and timely interventions. Targeted interventions for high-risk groups like youth, substance users and those with socio-economic challenges are necessary, alongside integrating mental health care and harm reduction into ART programmes. Addressing socio-economic barriers with financial, educational and social support improves access to care for marginalised populations. High-risk groups, including black individuals, MSM and youth, should receive community-based programmes and culturally competent care to improve HIV care engagement. Personalising ART regimens based on patient history and resistance testing optimises treatment outcomes, while regular screening for co-infections like TB and STIs helps maintain viral suppression and prevent VR.

Uninterrupted HIV care during and after incarceration is crucial for reducing treatment interruptions. Pre-travel counselling and support are important for maintaining adherence during travel. Ongoing research into the biological, behavioural and social factors of HIV VR is necessary to develop effective prevention strategies. Public health campaigns should raise awareness about ART adherence, regular healthcare visits and care engagement, focusing on at-risk populations and reducing stigma to improve HIV management and reduce VR.

## Conclusion

The study finds that the prevalence of HIV VR varies across different populations. Moreover, HIV VR is influenced by a complex interplay of behavioural, socio-economic, demographic, medical and environmental factors. While certain factors, such as ART regimen choices, alcohol use, treatment adherence, age, co-infections and DDIs, show consistent associations with VR, there is still much to learn about the relative importance of these variables and their interactions. Further research is necessary to disentangle these factors and to develop targeted interventions that address the diverse challenges faced by individuals living with HIV to reduce the risk of VR and improve long-term health outcomes.
